# New Evidence of *TLR4* and *TLR9* Variants Influencing Parasitaemia and Symptoms of *Plasmodium vivax* Infection

**DOI:** 10.1111/iji.70014

**Published:** 2025-09-18

**Authors:** Mikaele Monik Rodrigues Inácio Silva, Gabriela Maria Andrade Correia, Juliana Dal‐Ri Lindenau, Maristela Gomes Cunha, Maria Deise Oliveira Ohnishi, Ana Maria Revoredo Silva Ventura, Ândrea Ribeiro‐dos‐Santos, Mara Helena Hutz, Vinicius Albuquerque Sortica

**Affiliations:** ^1^ Programa de Pós‐graduação em Ciências da Saúde Universidade Federal de Alagoas Maceió Alagoas Brazil; ^2^ Departamento de Biologia Celular Embriologia e Genética Universidade Federal de Santa Catarina Florianópolis Santa Catarina Brazil; ^3^ Laboratório de Microbiologia e Imunologia Universidade Federal do Pará Belém Pará Brazil; ^4^ Programa de Ensaios Clínicos em Malária Instituto Evandro Chagas Sistema de Vigilância Sanitária Ministério da Saúde Belém Pará Brazil; ^5^ Laboratorio de Genética Humana e Médica Programa de Pós‐Graduação em Genética e Biologia Molecular Universidade Federal do Pará Belém Pará Brazil; ^6^ Departamento de Genética Universidade Federal do Rio Grande do Sul Porto Alegre Rio Grande do Sul Brazil

**Keywords:** Amazon region, Brazil, genetic variability, malaria, toll‐like receptor

## Abstract

Toll‐like receptors (TLRs) induce the production of pro‐inflammatory cytokines and regulate the immune response to *Plasmodium vivax* infection. Genetic variants of *TLRs* are associated with susceptibility and severity of malaria in different populations. This study aimed to evaluate the association between polymorphisms in *TLR1, TLR4, TLR7, TLR8, TLR9* and *TIRAP* and the clinical manifestations of malaria caused by *P. vivax* in a population from the Amazon region of Pará, Brazil. A total of 148 individuals with symptomatic uncomplicated malaria were genotyped for rs4833095, rs1927911, rs179008, rs3764880, rs352140 and rs8177374 variants, and their associations with parasitaemia levels, gametocytaemia and clinical index were analysed using generalised linear models. *TLR9* rs352140TT homozygotes had higher parasitaemia levels than C allele carriers (*p* = 0.034). *TLR4* rs1927911GG homozygotes had a higher clinical index than A allele carriers (*p* = 0.018). Our results describe, for the first time, the association of the rs1927911 variant found in intron 1 of *TLR4* with *P. vivax* malaria symptoms in Brazilian Amazonian population.

## 1 Introduction

Malaria is a febrile infectious disease caused by a protozoan of the genus *Plasmodium* that is transmitted to humans by the bite of female Anopheles mosquitoes (Aguiar et al. 2022). It is considered a serious public health problem worldwide, with more than 260 million cases reported in endemic countries in America, Africa, Asia and Oceania (WHO 2023). In Brazil, the prevalence of malaria is endemic in the Amazon region, comprising 99% of cases, of which the species *P. falciparum*, *P. malariae* and *P. vivax* are the main causes (Brasil 2024). In 2022, of all autochthonous cases, 84.2% (108,594) were caused by *P. vivax*, 13.9% (17,981) by *P. falciparum*, and 1.8% (2344) by mixed malaria infections (more than one parasite species at the same time). A total of 38 cases (< 0.1%) of malaria by *P. malariae* were recorded that year (SUS 2023; Brasil 2024). However, studies using real‐time PCR as a diagnostic tool demonstrated that *P. malariae* prevalence is underestimated in the malaria‐endemic Amazon region (Cunha et al. 2021). In Brazilian areas outside this region, most of the cases were imported from Amazonian states. However, some of the malaria cases were imported from other countries as Angola, South Africa, Mozambique, French Guiana, Guyana, Nigeria, Venezuela and Equatorial Guinea (Brasil 2022; Garcia et al. 2022).

Malaria is a complex disease affected by environmental and genetic factors, amongst which, the degree of immunity and genetic background are crucial for understanding the different impacts of diversity in response, progression and severity caused by clinical and physiological manifestations (Ramírez et al. 2023). In this context, studies have revealed the relationship between toll‐like receptor (TLR) variants involved in the immune response to malaria, which can influence clinical characteristics during pathogenesis (TLR1, TLR4 and TLR9) and severity (TLR2 and TLR6) (Mukherjee et al. 2019; Penha‐Gonçalves 2019; Antonelli et al. 2020; Dobbs et al. 2020). TLRs are a family of pattern recognition receptors (PRRs) that are important in the detection of pathogen‐associated molecular patterns (PAMPs) and those associated with cell damage (DAMPs). They play a key role in inducing the production of pro‐inflammatory cytokines, thereby activating transcription factor Nuclear Kappa B (NF‐κB) and bridging the gap between innate and adaptive immunity (Costa et al. 2017; Costa et al. 2018; Naing et al. 2021; Penha‐Gonçalves 2019; Ramirez Ramirez et al. 2022; Ramírez et al. 2023).

Genetic variants can significantly impact the expression and binding capacity of TLRs, altering their function and regulating their response to different pathogens. Single‐nucleotide variants (SNVs) of TLRs and Toll‐interleukin‐1 receptor domain‐containing adaptor protein (TIRAP) have been associated with malaria susceptibility and severity (Costa et al. 2017).

In addition to individual genetic variants, population structure and genomic ancestry significantly influence the interpretation of genetic associations. Previous studies indicate that genomic ancestry across different regions of Brazil is more uniform than expected (Cassiano et al. 2015; Pena et al. 2011); however, the extensive admixture amongst Europeans, Africans and Amerindians still necessitates careful control in association studies to prevent spurious results and accurately evaluate the impact of *TLR* polymorphisms (Cassiano et al. 2015).

Given the delicate interconnections between these components, it is essential to understand their pathophysiology, which can aid in the development of new strategies to reduce the morbidity of this disease. Therefore, this study aimed to analyse the potential influence of variants of *TLR1*, *TLR4, TLR7, TLR8, TLR9* and *TIRAP* on the clinical manifestations of malaria caused by *P. vivax* in the Amazon region of Brazil.

## 2 Materials and Methods

### 2.1 Subjects and Ethical Statement

We included 148 samples from individuals infected with *P. vivax* in the Amazon region of Pará in the study, as previously described (Sortica et al. 2012). Pará is a Brazilian state in the northern region of the country that borders the Brazilian states of Amapá, Amazonas, Maranhão, Tocantins, Mato Grosso, and Roraima, as well as the South American countries of Guyana and Suriname. All individuals were born in Pará, Brazil, and were diagnosed with uncomplicated symptomatic malaria. The diagnosis was made using thick blood film, and the levels of parasitaemia and gametocytaemia density per microlitre of blood were estimated by counting the number of parasites per 100 fields and double‐checked blindly by two expert microscopists. The clinical assessment was performed by doctors and recorded numerically as 0 (absent), 1 (mild), 2 (moderate), 3 (severe), and 4 (very severe). The most common symptoms were fever, chills, headache, arthralgia, lower back pain, asthenia, myalgia, dizziness, tinnitus, deafness, insomnia, nausea, vomiting, abdominal pain, eructation, flatulence, diarrhoea, choluria, oliguria, anuria, pallor, jaundice, pruritus, allergy, cough, dyspnoea, anorexia, splenomegaly and hepatomegaly. Blood samples from individuals diagnosed with malaria were taken just before the start of treatment. All patients received 1,500 mg of chloroquine combined with 210 mg of primaquine for 7 days, as recommended by the Ministry of Health (Brasil 2009). This project was approved by the Ethics Committees of the Evandro Chagas Institute (CEP/IEC‐0035) and Federal University of Pará (061/07 CEP‐CCS/UFPA). All the individuals included in the study signed an informed consent form. Participants younger than 18 years provided informed consent signed by parents to participate in the study.

### 2.2 Molecular Analysis

The samples were genotyped using Real‐Time Polymerase Chain Reaction (RT‐PCR) for the genetic variations of rs4833095, rs1927911, rs179008, rs3764880, rs352140, and rs8177374 of the *TLR1, TLR4, TLR7, TLR8, TLR9*, and *TIRAP* genes, respectively (Table 1). Applied Biosystems TaqMan SNP assays were used for this purpose. To establish genomic ancestry control, 48 molecular markers were used in the statistical analyses (Sortica et al. 2012).

**TABLE 1:** Description of the variants studied.

Gene	db SNP id	Position (GRCh38)	Alleles	Assay	Function
*TLR1*	rs4833095	chr04:38798089	C > T	C__44103606_10	missense
*TLR4*	rs1927911	chr09:117707776	A > G	C__11722141_10	intron
*TLR7*	rs179008	chrX:12885540	A > T	C___2259574_10	missense
*TLR8*	rs3764880	chrX:12906707	A > G	C___2183830_10	5 Prime UTR
*TLR9*	rs352140	chr03:52222681	C > T	C___2301954_20	synonym
*TIRAP*	rs8177374	chr11:126292948	C > T	C__25983622_10	missense

### 2.3 Statistical Analysis

Allelic and genotypic frequencies were estimated by direct counting, and deviations from the Hardy–Weinberg equilibrium were investigated using the chi‐square test. Principal component analysis was used to group symptoms into a clinical severity index. The *TLR* genotypes were associated with the levels of parasitaemia, gametocytaemia and clinical index using general linear models (GLMs). Parasitaemia and gametocyte levels were transformed to a logarithmic scale because they deviated from a normal distribution. However, the values transformed back into a standard scale were presented in the results as geometric means. In the GLMs, covariates of sex, age, African and European genetic ancestry, period of exposure and disease history were included because they are associated with malaria or correlated with levels of parasitaemia or gametocytaemia.

The statistical package SPSS 21.0 for Windows was used for statistical analysis. Significance was defined as a two‐tailed *p* value < 0.05, with Bonferroni correction. Effect sizes were calculated using Cohen's *d*, based on standardised differences between means (Fritz et al. 2011).

## 3 Results

### 3.1 Clinical Profile and Ancestry Indices

The individuals ranged in age from 12 to 88 years, with a median age of 32 years, and males accounted for 66.2% of the participants. The genetic ancestry of the group studied comprised an average proportion of 22.8% African, 41.0% European and 32.4% Native American ancestry (Table 2). The period of exposure of patients in the regions of infection was variable, with 53.4% of the population remaining in the area for between 1 and 25 days, 20.3% remaining in the area for more than 25 days, and 22.3% being residents of the endemic place of infection (Table 2). It was observed that 67.6% of the individuals were infected with *P. vivax* for the first time, 11.5% had malaria for the second time, and 20.3% had malaria more than twice. The individuals had infections with parasitaemia levels ranging from 50 to 75,000 parasites/µL at the time of diagnosis, and a median of 5500 parasites/µL. Sexual forms of *P. vivax* were observed in only 70 individuals at diagnosis, with a range of 15–4500 gametocytes/µL and a median of 100 (Table 2).

**TABLE 2:** Clinical characteristics of individuals infected with *P. vivax*.

Characteristics	
*N*	148 (100)
Sex	
Female	50 (33.8)
Male	98 (66.2)
Age	32 (12:88)
Genetic ancestry	
Amerindian	0.320 (0.109:0.695)
European	0.411 (0.199:0.708)
African	0.220 (0.094:0.516)
Parasitaemia	5.500 (50:75.000)
Gametocytaemia	100 (15:4.500)
Days in the infection site	
1–25	79 (53.4)
26 or more	30 (20.3)
residents	33 (22.3)
missing	6 (4.1)
Infection history	
first infection	100 (67.6)
second infection	17 (11.5)
third infection	30 (20.3)
missing	1 (0.7)

*Note*: Age, parasitaemia levels, gametocytaemia levels and genetic ancestry are presented as median (minimum:maximum). Sex, days at the infection site and infection history were presented as direct counts (relative frequency in %).

### 3.2 Allelic Frequency and Association of *TLR* Variants With Parasitaemia and Gametocytaemia

All polymorphisms were in Hardy–Weinberg equilibrium. No statistical difference was observed in the allele frequencies of these polymorphisms between sexes. Tables 3 and 4 show the allelic and genotypic frequencies of the *TLR* variants.

**TABLE 3:** Allelic and genotypic frequencies of the *TLR1*, *TLR4*, *TLR9*, and *TIRAP* polymorphisms.

Variant	Alleles		Genotypes		
*TLR1* rs4833095	C	T	CC	CT	TT
	147 (0.50)	147 (0.50)	38 (25.9)	71 (48.3)	38 (25.9)
*TLR4* rs1927911	A	G	AA	AG	GG
	127 (0.43)	169 (0.57)	32 (21.6)	64 (43.2)	52 (35.1)
*TLR9* rs352140	C	T	CC	CT	TT
	154 (0.52)	142 (0.48)	45 (30.8)	62 (42.5)	39 (26.7)
*TIRAP* rs8177374	C	T	CC	CT	TT
	263 (0.89)	33 (0.11)	118 (80.3)	26 (17.7)	3 (2.0)

*Note*: Data are presented as absolute frequencies (relative frequencies in %).

**TABLE 4:** Allelic and genotypic frequencies of *TLR7* and *TLR8* polymorphisms.

Gene	Alleles		Genotypes				
			Female			Male	
*TLR7* rs179008	A	T	AA	AT	TT	A	T
	238 (0.81)	56 (0.19)	36 (72.0)	13 (26.0)	1 (2.0)	76 (78.4)	21 (21.6)
*TLR8* rs3764880	A	G	AA	AG	GG	A	G
	161 (0.55)	131 (0.45)	19 (38.8)	21 (42.9)	9 (18.4)	51 (52.6)	46 (47.4)

*Note*: Data presented as absolute frequency (relative frequency in %).

An analysis using GLM was performed to test the association between genetic variants and parasitaemia levels in patients. The models were adjusted for sex, age, African and European genetic ancestry, exposure periods and disease history. Homozygotes for the T allele of the rs352140 polymorphism of *TLR9* showed higher parasitaemia than carriers of the C allele (*p* = 0.034) (Table 5). This analysis presented a power of 57% and a medium effect size (*d* = 0.32), considering Cohen's classification (Fritz et al. 2011). No other variants were significantly associated with parasitaemia levels in this analysis. The association between the patients' gametocytaemia levels and the genetic variants of *TLRs* and *TIRAP* was tested using a GLM controlled by the same parameters. There was no association between the variants and gametocyte levels in individuals with *P. vivax* malaria (Table 6).

**TABLE 5:** Parasitaemia level and clinical index according to TLRs and TIRAP genotypes by a genetic recessive model.

Variants	Genotypes	*N*	Mean parasitaemia	*p* value	Cohen's *d*	Clinical index	*p* value	Cohen's *d*
*TLR1* rs4833095	CT + TT	104	4634.47 (3140.51:6823.39)	0.307		−0.034 (−0.279:0.211)	0.404	
	CC	35	6266.14 (3564.51:11,015.39)			−0.190 (−0548:0.168)		
*TLR4* rs1927911	AA+AG	90	4709.77 (3118.89:7095.78)	0.546		−0.211 (−0.462:0.039)	**0.018**	0.34
	GG	49	5533.50 (3388.44:9057.33)			0.184 (−0.124:0.492)		
*TLR7* rs179008	AT + TT	34	6982.32 (3944.57:12,359.47)	0.140		−0.124 (−0.486:0.238)	0.720	
	AA	105	4487.45 (3047.89:6591.74)			−0.056 (−0.301:0.189)		
*TLR8* rs3764880	AG + GG	70	5260.17 (3419.79:8072.35)	0.483		−0.132 (−0.398:0.134)	0.401	
	AA	68	4385.31 (2747.89:6998.42)			0.004 (−286:0.293)		
*TLR9* rs352140	CC + TC	100	4207.27 (2851.02:6208.69)	**0.034**	0.32	−0.107 (−0.356:0.143)	0.475	
	TT	38	7762.47 (4528.98:13,304.54)			0.026 (−0.324:0.375)		
*TIRAP* rs8177374	CT + TT	28	5296.63 (2904.02:9660.51)	0.057		−0.030 (−0.408:0.349)	0.779	
	CC	111	4897.79 (3280.95:7311.39)			−0.087 (−0.337:0.162)		

*Note*: Parasitaemia levels were presented as geometric means (95% CI) and calculated by a general linear model using sex, age, African and European genetic ancestry, period of exposure and infection history as covariates. The clinical index was presented as geometric mean (95% CI) and calculated by the general linear model using sex, age, African and European genetic ancestry, exposure period, disease history and parasitaemia as covariates.

**TABLE 6:** Association between gametocyte levels and *TLR* and *TIRAP* genetic variants.

Variants	Genotypes	*N*	Mean gametocytaemia	*p* value
*TLR1* rs4833095	CT + TT	47	178.39 (106.27:299.47)	0.635
	CC	22	154.78 (86.75:275.89)	
*TLR4* rs1927911	AA + AG	39	176.09 (104.17:297.67)	0.725
	GG	30	159.33 (91.74:277.00)	
*TLR7* rs179008	AT + TT	17	164.51 (84.35:321.18)	0.926
	AA	52	169.52 (104.48:275.34)	
*TLR8* rs3764880	AG + GG	32	149.61 (90.92:245.92)	0.982
	AA	36	148.56 (85.03:259.82)	
*TLR9* rs352140	CC + TC	46	198.54 (116.75:337.65)	0.232
	TT	22	136.18 (76.48:242.74)	
*TIRAP* rs8177374^a^	CT	17	162.23 (80.48:327.01)	0.891
	CC	52	170.72 (103.34:282.31)	

*Note*: General linear models using sex, age, African and European genetic ancestry, period of exposure and medical history as covariates. Gametocytaemia levels are presented as the geometric mean (95% CI).

^a^ *P. vivax* gametocytes were present only in individuals genotyped as TIRAP rs8177374 CT or CC.

### 3.3 Association of *TLR* Variants With Clinical Severity Index

Principal component analysis was performed to group the symptoms presented by patients at the time of diagnosis. Symptoms were evaluated using numerical scores ranging from zero to four (absent, mild, moderate, severe and very severe). In the principal component analysis, the first component explained 39.3% of the variability in patients' symptoms. The first component identified the highest weights for the symptoms of fever, headache, chills, myalgia, arthralgia, lower back pain, abdominal pain, asthenia, dizziness, nausea and anorexia.

The first component was used as a clinical index in the GLM to investigate the association between genotypes and the severity of uncomplicated symptomatic malaria caused by *P. vivax*. Age, sex, exposure period, disease history, European and African genetic ancestry and parasitaemia were used as covariates in the analysis (Table 5).

Patients with homozygous *TLR4* rs1927911GG had a higher clinical index than those carrying the A allele (*p* = 0.018). This analysis presented a medium effect size (*d* = 0.34), considering Cohen's classification (Fritz et al. 2011), and a power of 66%. Polymorphisms in the *TLR1, TLR9, TLR7, TLR8* and *TIRAP* genes were not associated with the clinical index (Table 5).

## 4 Discussion

The immune response to malaria has different aspects, which can be partially explained by molecular pathways in which variations in the genetic background can modulate the immune response of patients affected by this disease. Thus, the combination of these variants can result in compromised or effective immune responses during infection (Mukherjee et al. 2019; Penha‐Gonçalves 2019; Antonelli et al. 2020; Dobbs et al. 2020).

*TLR9* has two exons and is located in the 3p21.3 region of the short arm of chromosome 3. TLR9 interacts only with intracellular ligands because of its location in endosomes (Krishnegowda et al. 2005). Polymorphisms in the promoter region of this gene are associated with alterations in the expression of this protein, which have different consequences for the modulation of cytokines and immune responses. In our study, *TLR9* rs352140 TT homozygotes were associated with higher levels of parasitaemia in *P. vivax* infection. These findings are consistent with those of Omar et al. (2012), who associated this variant with an increased risk of symptomatic malaria and higher levels of parasitaemia in *P. falciparum* in a Ghanaian population. Another study conducted in Colombia showed that *TLR9* rs352140 CC homozygotes are protected against malaria infections (Mario‐Vásquez et al. 2021). This result is consistent with the finding that TT homozygotes are more susceptible to malaria and have higher levels of parasitaemia.

*TLR4* is located in the 9q32‐q33 extended arm region of chromosome 9. This gene has four exons and is highly expressed in lymphocytes, monocytes, neutrophils and splenocytes. TLR4 interacts with intra‐ and extracellular ligands because it is located on the surfaces of cells and endosomes. Variants in *TLR4* have been described to alter its function and modulate the response to different infections (Dhangadamajhi et al. 2017; Rani et al. 2018). *TLR4* rs4986790, rs4986791 and rs5030719 have been associated with modulation of the expression of the cytokine cascade in *Plasmodium* spp. malaria, with levels of parasitaemia and susceptibility in symptomatic cases of clinical *P. vivax* malaria (May et al. 2010; Rani et al. 2018; Sirisabhabhorn et al. 2021; Ramírez et al. 2023). Recently, children infected with *P. falciparum* from Nigeria, heterozygotes for the rs4986790 and rs4986791 variants, were associated with a higher susceptibility to complicated malaria (Bamikole et al. 2025). In our study, the *TLR4* rs1927911 variant was associated with the clinical malaria index levels in an unprecedented manner. The rs1927911 variant is found in intron 1 of *TLR4* and is associated with the risk of asthma, atherosclerosis and different types of cancer (Vawda et al. 2014; Xie et al. 2017; Moura et al. 2022). The effect of this polymorphism on *TLR4* expression and function has yet to be determined. Functional studies may help to clarify the role of this variant in modulating the immune response to infectious diseases. The association of *TLR9* rs352140 polymorphism with parasitaemia and the clinical presentation of malaria in the admixed population of Pará in this study corroborates the data found in the literature associating different variants of this gene with the severity and parasitaemia of *Plasmodium* spp. infections in populations of different ethnic origins (Omar et al. 2012; Mario‐Vásquez et al. 2021; Naing et al. 2021).

Furthermore, meta‐analyses (Dhangadamajhi et al. 2017; Naing et al. 2021; Ramirez Ramirez et al. 2022) and recent studies (Bamikole et al. 2025) show great variability in the genetic models included in statistical analyses, in the populations and in the age range of the individuals studied. These studies showed that *TLR9* rs5743836 is associated with the risk of severe malaria in Indian adults with *P. falciparum* by stratified analysis (Dhangadamajhi et al. 2017) and associated with malaria (*P. falciparum* and *P. vivax*) severity in a heterozygous model and with a subgroup of adults from Asian countries under this model in a stratified analysis (Naing et al. 2021). *TLR9* rs187084 was associated with severe *P. falciparum* malaria in allelic and homozygous models (Dhangadamajhi et al. 2017) and malaria severity in a heterozygous model and in a subgroup of children of non‐Asian countries under an allelic model in the stratified analysis (Naing et al. 2021). Another study showed that this variant was associated with the development of uncomplicated *P. falciparum* malaria in a heterozygous model (Bamikole et al. 2025). The meta‐analysis performed by Dhangadamajhi et al. (2017), based on Indian adult populations, and Naing et al. (2021), which included a subgroup of adults from several Asian countries, showed that *TLR4* variants rs4986790 and rs4986791 have no statistically significant association with susceptibility and severity of malaria. However, a more recent meta‐analysis showed an association with parasitaemia for *TLR1* in African populations*, TLR4* in Indian populations and *TLR9* in cohorts from Africa, India and the Brazilian Amazon. In addition, *TLR2* and *TLR6* genes were associated with malaria severity in African populations, but not with susceptibility under different conditions (Ramirez Ramirez et al. 2022).

The above scenario describes the complexity of studies, such as the present study, with a structured admixed population. The gaps arising from the divergence of significant associations between genetic models and their effect on severity and susceptibility may reflect ancestral population backgrounds, in which ancestry carries significant weight in the genetic distribution of the population (Mario‐Vásquez 2020; Ramirez Ramirez et al. 2022).

In our study, no associations were found between the rs4833095, rs179008, rs3764880 and rs8177374 polymorphisms in the *TLR1, TLR7, TLR8* and *TIRAP* genes and parasitaemia, gametocytaemia or the clinical malaria index. These data are consistent with those of Costa et al. (2017), who studied the genetic susceptibility of *P. vivax* in the Brazilian Amazon population. Due to the small sample size, we carried out genetic analyses using only the recessive model, which seems to be more robust than the additive model in GWAS‐type genetic study simulations, with better accuracy, precision and discrimination in identifying disease‐risk SNPs (Liu et al. 2021). The results of our study reinforce the importance of variants in the *TLR4* and *TLR9* genes in modulating the immune response to *P. vivax* malaria.

The complexity of host–parasite interactions in *P. vivax* malaria should also be interpreted in the context of the genetic diversity of the parasite itself. Current evidence indicates that *P. vivax* populations circulating in the Brazilian Amazon are not clonal and instead exhibit substantial genomic variability, which results in multiple distinct lineages across the region (Ibrahim et al. 2023). Moreover, population‐based genomic studies have identified Brazil‐specific mutations in genes related to mosquito transmission stages and antimalarial drug response, which may influence infection dynamics and clinical outcomes (Ibrahim et al. 2023). Similarly, mitochondrial genome analyses have documented extensive haplotype diversity and divergent evolutionary lineages in South America, including Brazil (Taylor et al. 2013). This parasite diversity, coupled with host genetic variability, may influence parasite virulence, immune evasion strategies and consequently the host immune response, potentially contributing to heterogeneity in clinical outcomes and in the associations observed with *TLR* polymorphisms. Considering both host and parasite genetic backgrounds is therefore essential for a comprehensive understanding of susceptibility to malaria and disease progression (Cornejo et al. 2015; Ibrahim et al. 2023).

However, our study has some limitations: (1) the small sample size made it impossible to carry out gene–gene interaction analyses; (2) due to the freezing of the blood samples before genotyping, it was not possible to measure other biological markers, such as cytokine levels, to corroborate the findings of the analyses with the genotypes; (3) the sample consisted only of cases of uncomplicated symptomatic malaria, and the results may not represent the same significance in asymptomatic individuals or those with complicated malaria. Despite these limitations, our findings indicate that variants of *TLR4* and *TLR9* may be molecular markers for the clinical presentation of malaria caused by *P. vivax* in the admixed population of the Brazilian Amazon. In contrast, a recent study conducted on the Brazil–French Guiana border, analysing 76 individuals, reported associations between *TLR4*, *TLR6* and *TLR9* polymorphisms and parasitological parameters, including gametocyte levels (Cerilo‐Filho et al. 2025). This difference likely reflects the smaller sample size and distinct population structure of the Cerilo‐Filho cohort, as well as regional variability in host genomic ancestry and parasite genetic diversity. Complementary studies with new samples and additional biological variables should be conducted to replicate these findings and help understand the variation in the presentation of symptomatic *P. vivax* infections.

## 5 Conclusions

The results of our study reinforce the importance of variants in the *TLR4* and *TLR9* genes in modulating the immune system's response to *P. vivax* malaria. Our results describe, for the first time, the association of the rs1927911 variant found in intron 1 of *TLR4* with *P. vivax* malaria symptoms. These results highlight the importance of continuous studies of genetic variants in the susceptibility and severity of infectious and neglected diseases in the admixed population in Brazil. This will advance the knowledge of the pathophysiology of these diseases and help develop diagnostic and prevention strategies.

## Ethics Statement

This study was conducted in accordance with the Declaration of Helsinki and approved by the Ethics Committees of the Evandro Chagas Institute (CEP/IEC‐0035/09 and CAAE 0038.0.072.000‐09) and Federal University of Pará (CEP‐CCS/UFPA 061/07).

## Consent

Informed consent was obtained from all subjects involved in the study or their legal guardian.

## Conflicts of Interest

The authors declare no conflicts of interest.

## Data Availability

The data that support the findings of this study are available from the corresponding author upon reasonable request.
